# The Assessment of Myocardial Longitudinal Strain in a Paediatric Spanish Population Using a New Software Analysis

**DOI:** 10.3390/jcm11123272

**Published:** 2022-06-08

**Authors:** Cristhian H. Aristizábal-Duque, Juan Fernández Cabeza, Isabel María Blancas Sánchez, Mónica Delgado Ortega, Pilar Aparicio Martinez, Manuel Romero-Saldaña, Francisco Javier Fonseca del Pozo, Manuel Pan, Martin Ruiz Ortiz, María Dolores Mesa-Rubio

**Affiliations:** 1 Cardiology Department, Reina Sofia University Hospital, Maimonides Institute of Biomedical Research of Cordoba (IMIBIC), 14004 Cordoba, Spain; 2 Urgency Department, Reina Sofia University Hospital, Maimonides Institute of Biomedical Research of Cordoba (IMIBIC), 14004 Cordoba, Spain; 3 Department of Nursing, Pharmacology and Physiotherapy, Faculty of Medicine and Nursing, Maimonides Institute of Biomedical Research of Cordoba (IMIBIC), University of Córdoba, 14004 Cordoba, Spain; 4 Córdoba and Guadalquivir Health District, Reina Sofia University Hospital, Maimonides Institute of Biomedical Research of Cordoba (IMIBIC), 14004 Cordoba, Spain

**Keywords:** Ehocardiography, child, ventricular function, myocardial strain, speckle-tracking

## Abstract

Background: Two-dimensional speckle-tracking echocardiography (2DSTE) has been present for years. However, it is underutilized due to the expertise and time requirements for its analysis. Our aims were to provide strain values in a paediatric Spanish population and to assess the feasibility and reproducibility of a new strain software analysis in our environment. Methods: A cross-sectional study of 156 healthy children aged 6 to 17 years. Longitudinal strain (LS) analysis of the left ventricle, right ventricle, and left atrium was performed. Feasibility and reproducibility were assessed. The associations of clinical and echocardiographic variables with strain values were investigated by multivariate analysis. Results: Mean age was 11 ± 3 years (50% female). Feasibility of LS measurement ranged from 94.2% for left ventricle global LS (LVGLS) to 98.1% for other chamber strain parameters. Strain values were 26.7 ± 2.3% for LVGLS; 30.5 ± 4.4% and 26.9 ± 4% for right ventricle free wall LS (RVFWLS) and four chambers view LS (RV4CLS) respectively; and 57.8 ± 10.5%, 44.9 ± 9.5%, and 12.9 ± 5.5% for left atrium LS reservoir phase (LALSr), conduct phase (LALScd) and contraction phase (LALSct), also respectively. Body surface area (BSA) and age presented a negative correlation with strain values. Higher values were found in females than in males, except for LALScd. Excellent intra- and inter-observer reproducibility were found for right and left ventricular strain measurement, with intraclass correlation coefficients (ICC) ranging from 0.88 to 0.98, respectively. In conclusion, we described strain values in a healthy Spanish paediatric population. LS assessment by this new strain analysis software by semi-automatic manner was highly feasible and reproducible.

## 1. Introduction

The assessment of cardiac function requires continuous advances. There is great interest in finding a reliable, reproducible and easily accessible tool to assess both systolic and diastolic function [1]. Myocardial deformation by strain is a novel method in echocardiography which could perfectly meet these objectives [2,3]. 

Currently, its most widespread use is the assessment of longitudinal strain by 2DSTE [4]. However, this technique is less used than it should be because its availability is generally limited to specialized centres owing to the need for training and the time required to perform it. Therefore, artificial intelligence, either semi- or fully automatic, has been developed [5,6]. 

Myocardial strain evaluation in the paediatric population is of growing interest. Normal strain values have been reported by different authors for the left ventricle [7,8,9,10,11,12], right ventricle [13,14,15] and left atrium [16,17,18], but none have studied a Spanish paediatric population. Variation between countries has been reported for left ventricular volumes and to a lesser extent for left ventricular ejection fraction and strain values [19]. 

Most data have been reported in an independent manner for each cardiac chamber, using various methodologies, different equipment, and even different parameters in their report; this is understandable since most papers are prior to the publication of the current recommendations [20,21]. Our aims were to provide strain values for the left ventricle, the right ventricle, and the left atrium in a paediatric Spanish population and to assess the feasibility and reproducibility using a new strain analysis software according to current recommendations.

## 2. Materials and Methods

### 2.1. Study Population

The research was structured as a prospective, cross-sectional descriptive study. It was conducted in a locality of 2864 inhabitants in the south of Spain between March 2017 and April 2018. Participation was offered freely in all the educative institutions of the city and to all its students.. The main exclusion factors were pre-existing cardiopulmonary or systemic diseases, including congenital diseases and such cases detected during the study; first-degree family history of sudden cardiac death (SCD) or diagnosed cardiomyopathy. The age limit was 17 years old. 

Anthropometric measurements and blood pressure were taken prior to the echocardiographic study. The Haycock formula was used for body surface area [22]. Participants who met the WHO criteria for obesity according to age-adjusted body mass index and hypertension (HT) were excluded from the analysis of normality. Every measurement was taken in accordance with current recommendations [23].

### 2.2. Conventional Echocardiographic Study

All studies were performed with the same echocardiography equipment iE33 (Philips Medical Systems, Amsterdam, The Netherlands) with the S5-1 probe 1–5 MHz. The images were taken in parasternal long and short axis, apical, subcostal and suprasternal views, as well as in non-conventional views when necessary. The images were digitally stored as raw data and analysed off-line in Q Station 3.7 for conventional measurements. These analyses included dimensions, volumes and systolic and diastolic function and followed the current recommendations for studies in paediatric populations [24,25]. 

### 2.3. Strain Analysis

For the study of myocardial deformation, digital acquisition of at least three cardiac cycles was performed in the apical two-, three- and four-chamber views for the left ventricle. A focused apical view for the right ventricle and left atrium was also acquired for both. Equipment settings, such as sector width and depth, were optimized to obtain an image frequency of at least 60 Hz. All images were acquired by two expert echocardiographers (CAD and JFC).

Strain analysis was performed with the new Q-station, version 13.0 of Qlab (Philips Medical Systems, Amsterdam, the Netherlands), which integrates the TOMTEC auto-strain software and has been designed according to the new recommendations for the assessment of longitudinal strain. 

Whilst the longitudinal deformation, except for LALSr, gives negative values, in order to a better understanding of the data, we will express the measurements in absolute numbers, as proposed by Flashkampf, FA et al. [26], parting from the assumption that the higher the value, the better the strain function and vice versa.

**Figure 1:**
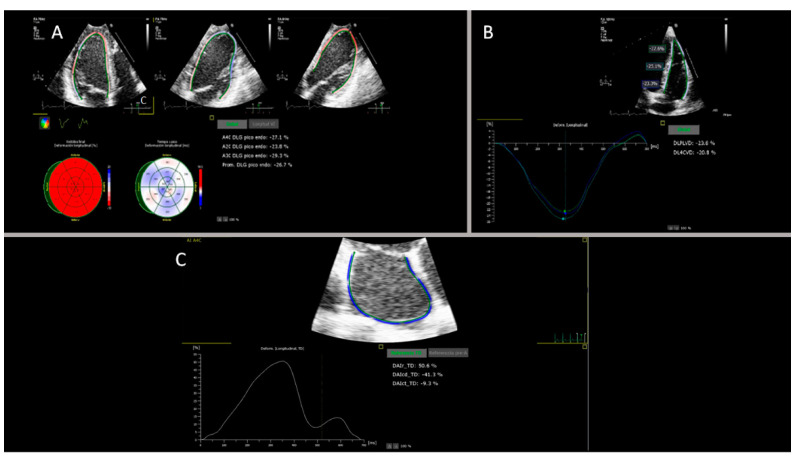
Panel (**A**): Output of the left ventricular longitudinal strain analyses. The upper part shows, from left to right, the endocardial tracking of chambers four, two and three; the bottom left part shows the bull’s eye with the strain values (%) and the time to peak longitudinal strain (ms) for the 18 segments. On the bottom right part, the LVGLS results for chambers four, two and three and the average LVGLS (%) can be found. Panel (**B**): Output of the right ventricular longitudinal strain analyses. The upper part shows the endocardial tracing in the four apical chambers: basal, medial, apical and RVFWLS: (%). The bottom left part shows the deformation curves versus the time for each segment (coloured curves). On the right, the results of the average RVFWLS and the average RV4CLS (%) are found. Panel (**C**): Output of the left atrium longitudinal strain analyses. At the top is the apical four-chamber endocardial tracing; at the bottom left is the strain versus time curve, and at the bottom right, the GLS results for each of the phases of the atrium (reservoir, conduit and contraction) (%) can be found.

Left Ventricle: The three left ventricle apical windows were selected according to the vendor specifications, and the left ventricle auto-strain function was activated. After the labelling of the phases and tracking, which was performed automatically by the software, manual correction was added when appropriate (Figure 1A).

Right Ventricle: A focused image of the right ventricle was selected. Software analysis was performed specifically for this chamber. The right ventricle auto-strain function was activated. After automatic tracking and analysis by the software, the borders were manually corrected when necessary, and two results were provided: an average free wall view and a four-chamber view, including the septum segments (Figure 1B). For left and right ventricle analysis, the peak systolic strain was chosen. 

Left atrium: An apical four-chamber window focused on the left atrium was used. As for the right ventricle, the analysis software is specific to this chamber. The left atrial automated strain was activated, and the system provided the direct analysis of the three phases of the atrium: reservoir, conduit and atrial contraction, which were manually corrected if necessary (Figure 1C). For the cycle reference, the R-R methodology was chosen because it is the most reported.

### 2.4. Statical Analysis

The normality of the data distribution was assessed using the Kolmogorov-Smirnov method. Continuous variables were expressed as mean and standard deviation (SD) or as median and interquartile ranges for variables with a non-normal distribution, whereas proportions data were expressed as frequencies and percentages. For the comparison of subgroups, ANOVA was used for quantitative variables with Bonferroni or Games-Howell post hoc analysis as appropriate, and the chi-square test was used for qualitative variables. The associations of strain values with the different anthropometric, echocardiographic and sex variables were evaluated by univariate regression analysis and multivariate models for those with a significance *p* < 0.01. The feasibility of the strain measures was expressed in percentages, estimated as a ratio of the number of interpretable cases to the number of assessed cases, multiplied by one hundred.

The intra and inter-observer variability assessment was performed by combining the intraclass correlation coefficient and Bland-Altman plots. To assess intra-observer variability, the main analyst (CAD) randomly repeated the analysis on 20 random cases three months after the previous analysis and blinded the previously obtained values. As for the establishment of the inter-observer variability, an additional 20 different cases were randomly selected, and another echocardiographer (JFC) independently analysed the data in a blinded manner with respect to the values obtained by the main analyst. SPSS for Windows, version 21.0 (SPSS, Inc., Chicago, IL, USA) was used for the analysis, (https://www.ibm.com/analytics/spss-statistics-software, accessed on 2 June 2022), and a *p* < 0.05 was considered significant.

## 3. Results

From the 265 children and adolescents who were initially eligible, 26 decided not to participate for personal reasons; 49 met criteria for obesity with or without HT and therefore were excluded; 8; were studied for isolated HT, the cases for 13 could not be analysed by this software due to technical problems leading to failure in the recording. 8 were diagnosed with cardiac pathologies during the echocardiographic study, and 4 were ≥18 years old and were excluded according to the age criterion. The final sample consisted of 156 subjects, and 78 (50%) were females. The mean age was 11 ± 3 years. Significant differences were found between males and females in systolic blood pressure (SBP), basal right ventricular diameter, left atrial volume, left ventricular end-diastolic volume and other conventional echocardiographic parameters, although all values were within normal limits. Table 1 shows the main characteristics of the population categorized by sex.

The samples were analysed by age group using terciles, which proved to be the most homogeneous form of distribution for our sample. As expected, significant differences were found in most of the anthropometric and echocardiographic variables, except for left ventricular ejection fraction (LVEF), mitral A velocity and TEI index. Table 2 shows the distribution of the conventional echocardiographic variables categorized by age group.

### 3.1. Feasibility Analysis

The feasibility of all cardiac chamber strain analyses was studied by age group and sex. For the entire sample, it ranged from 94.2% for the left ventricle to 98.1% for several strain parameters of the right ventricle and the left atrium. In the categorized analysis, it ranged from 87.5% for the left ventricle in the age group 10 to 12 years and up to 100% for all variables except for the left ventricle in the age group 6 to 9 years. Table 3 summarizes the distribution of the feasibility.

### 3.2. Effect of Sex, Age and Body Surface Area on Strain Values

**Figure 2:**
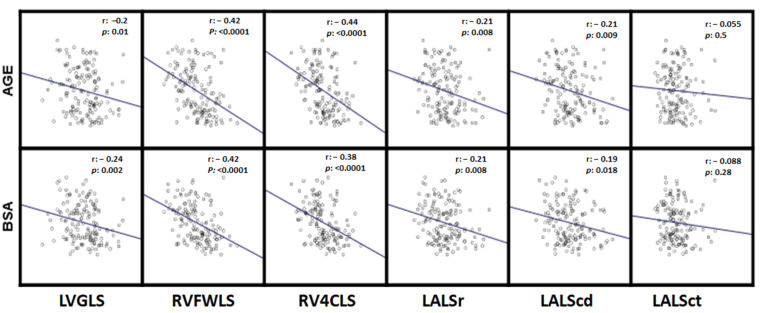
Scatter plots for correlations of strain values with age and BSA. Body surface. r, The Pearson correlation coefficient; the regression slope is displayed in blue. LVGLS: Left ventricle global longitudinal strain, RVFWLS: Right ventricle free wall longitudinal strain, RV4CLS: Right ventricle four chamber longitudinal strain, LALSr: Left atrial longitudinal strain reservoir phase, LALScd: Left atrial longitudinal strain conduct phase, LALSct: Left atrial longitudinal strain contraction phase.

Table 4, Table 5 and Table 6 show the strain values of the different cardiac chambers, categorized by sex, age group and body surface area (BSA). Figure 2 shows the bivariate correlations between the strain and the different cardiac chambers with age and BSA.

Average strain values were significantly higher in female than male subjects for all variables except for LALScd (Table 4). We also found a significant tendency of lower mean strain values with higher age, except for LALScd (Table 5, Figure 2).

Finally, higher BSA was inversely related to mean strain values for most variables in bivariate correlations (Figure 2), even though these associations were only significant for right ventricle strain and LALSr when BSA was categorized in tercile groups (Table 6). 

### 3.3. Variables Independently Associated with Strain Values

#### 3.3.1. Left Ventricle

In addition to age, sex and BSA, LVGLS was significantly associated with SBP, left ventricle mass, left ventricle diastolic diameter (DDLV), end diastolic volume and left ventricle ejection fraction (LVEF). In the multivariate regression model, only EDV and LVEF were independent predictors of LVGLS. However, an extremely low coefficient of determination (R^2^: 0.091) was observed for the model. The results are summarized in Table 7.

#### 3.3.2. Right Ventricle

Several variables presented a significant association with RVFWLS: age, sex and BSA, previously mentioned, and SBP, LV mass, DDLV, EDV, right ventricle (RV) basal, right ventricle diastolic area (RVDA) and TAPSE. 

In the multivariate regression model, only BSA, LV mass and sex were independently associated with RVFWLS. A poor coefficient of determination (R^2^: 0.276) was also observed. 

Regarding the RV4CLS, other variables were significantly associated within the univariate analysis in addition to age, sex and BSA: LV mass, DDLV, EDV, RV basal, A vel and left atrium volume (LA vol). In the multivariate analysis, only age and sex were independent predictors, with a low coefficient of determination (R^2^: 0.227) for the final model. The results are summarized in Table 8.

#### 3.3.3. Left Atrium

LALSr was significantly associated with DDLV, EDV, RV basal, RVDA, A Vel and LA vol in univariate analysis. Only LA vol remained as an independently predictor of this variable in the multivariate regression model, and it showed a very low R^2^: 0.083.

The variables that were significantly associated with LALScd in univariate analysis were LV mass, DDLV, EDV, RV basal and LA vol. The only independent predictor in multivariate analysis was LV mass, also with a very low coefficient or determination for the model (R^2^: 0.066).

Finally, LALSct was associated with sex, RVDA, A vel, tissue doppler velocity (TDV) A septal and E/A ratio. Only A velocity was independently associated with this variable in the multivariate regression model, also with a low coefficient or determination for the model (R^2^: 0.067). The results are summarized in Table 9.

### 3.4. Reproducibility

**Figure 3:**
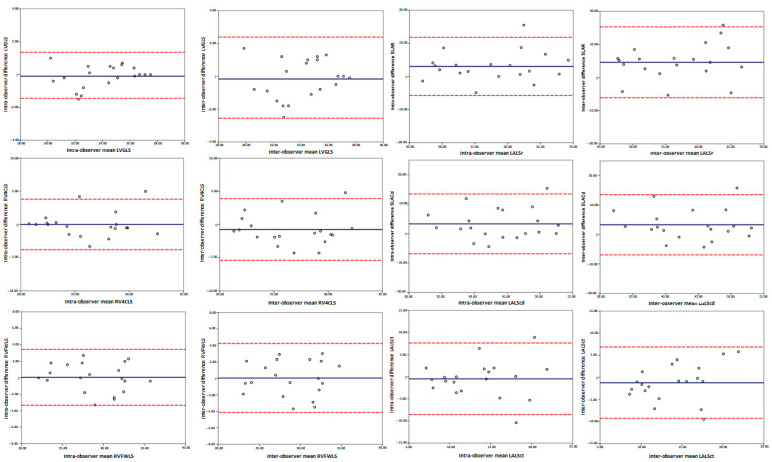
The Bland-Altman plots of the intra- and inter-observer measurements. LVGLS: Left ventricle global longitudinal strain, RVFWLS: Right ventricle free wall longitudinal strain, RV4CLS: Right ventricle four chamber longitudinal strain, LALSr: Left atrial longitudinal strain reservoir phase, LALScd: Left atrial longitudinal strain conduct phase, LALSct: Left atrial longitudinal strain contraction phase.

Both intra- and inter-observer reproducibility were excellent for the right and left ventricle strain parameters, with ICCs ranging from 0.88 to 0.98, and good for the left atrial strain parameters, with ICCs ranging from 0.72 to 0.86 (Table 10). The Bland–Altman plots in Figure 3 show the absence of proportional bias.

## 4. Discussion

In this study, we assess the feasibility of strain analysis of the left ventricle, right ventricle and left atrium in a population of healthy children and adolescents without structural heart disease through semi-automatic analysis using the most recent version of a commercially available platform. We provide strain values for the three cardiac chambers as well as their associations with different clinical and echocardiographic variables.

The main findings of the present study are the following. First, longitudinal strain by 2D STE analysis of the different cardiac chambers is highly feasible and reproducible with the use of this new software. Second, there are modest but significant associations of numerous anthropometric and conventional echocardiographic variables with the strain of the different cardiac chambers.

The use of strain has numerous theoretical advantages over conventional echocardiography parameters [27]. It shows potential use in multiple pathologies, including congenital heart disease [28,29,30,31]. Additionally, 2DSTE has multiple published advantages that are incorporated into clinical practice and that currently stand out in oncology patients [32], who unfortunately include paediatric populations who could benefit greatly from this study.

However, routine use of the technique has been limited by the time required to perform the analysis and the expertise necessary for its application. It is in these cases that artificial intelligence has been used and semi- or fully automatic cardiac function systems have been developed [5,6,33].

We have used the latest version of QLAB from Philips^®^, which integrates the TOMTEC auto-strain software. As a result of our usual practice and as already described by Kawakami et al. [5], the auto-strain software requires minor corrections in the endocardial border in a high number of cases. Therefore, we decided to perform the analysis using the semi-automatic setting from the beginning when planning the study. It was not considered necessary to provide differences between the automatic and semi-automatic methods, as the automatic method proved to be less reliable.

Our results stand out for their high feasibility and intuitive handling, which we believe could potentially help the routine implementation of strain analysis in a larger number of centres caring for paediatric and congenital cardiac pathologies.

Several similar studies have investigated the association between left and right ventricular strain and age and found negative correlations [7,34,35,36,37]. Our findings are similar to those reported by Cantinotti et al., unsurprisingly since their study has the largest sample size to date, although they used an older workstation from our same vendor. However, this relationship is quite weak, being only slightly stronger for the right ventricle, where age remains an independent predictor. This finding differs from results from Levy et al. [14], who failed to find such an association. On the other hand, the normal values for the right ventricle and the higher values in the free wall than in the four chambers in their study coincide with our findings.

It is noteworthy that we found, although only weakly, a difference in strain by sex, with greater strain in females than in males. This relationship is an independent factor in both parameters of right ventricular strain. Cantinotti et al. also reported sex differences for right ventricle strain in favour of females in the age group ranging from 11 to 18 years. Moreover, in the young adult population, Park et al. [15] for the right ventricle describe such a finding, which they theorize could be due to an estrogenic hormonal effect.

With regard to the left atrium, the predictor for the reservoir phase was the left atrial volume; for the conduit phase, the left ventricular mass; and for the contraction phase, the velocity of the mitral filling A wave. This is related, in accordance with previous groups that already correlated left atrial strain values, to the doppler echocardiographic parameters for the same phase of the atrial cardiac cycle function, probably because it is highly influenced by left ventricular compliance as well as by atrial size and volume [38,39,40]. As previously reported, we theorized that this finding supports the idea that left atrial strain analysis could be a good tool for simultaneously evaluating left atrial systolic and ventricular diastolic function.

It is notable that our strain results are slightly higher than those reported with General Electric (GE), in agreement with the values reported by Cantinotti et al., who have the largest sample and developed their study using the Philips platform; our values are practically the same as theirs in the comparable age groups. This strongly suggests that there are still inter-vendor differences, at least with the main samples reported in this age group.

Our research aim is to show the possibility of assessing strain in the paediatric population in a user-friendly way and a replicable manner using currently available methods. We are convinced that the strain values that we have reported can add value to the already growing information on strain in children and adolescents that is currently available. The findings can encourage other groups to make use of this or similar methods and include them in their daily use of strain cardiac function a reality.

To the best of our knowledge, our data represent the first report of strain values in school-aged paediatric population in our environment obtained with this new software according to the latest strain analysis recommendations.

### Limitations

Given the free nature and the participating centres, the sample contains a small number of subjects from a single geographic region and common ethnicity. Our sample did not include children under 6 years of age. No neonates or young children were included. We did not make any exclusions based on ethnic origin, which differs from other studies that have done so. We used only one vendor for the analysis (Philips^®^). However, at the time of this study, there was no other commercial platform available with specific analysis software for each cardiac chamber apart from the one we used in this study. Recently, other commercial vendors have made available similar tools to the one we used, and comparisons between them should be the subject of future study.

In addition, even though we acquired short-axis images for circumferential and radial strain analysis, to date the platform does not have specific semi-automatic or automatic software for this purpose. Therefore, it was decided not to include this in the study.

Another limitation is that left atrial strain could only be performed in the apical four-chamber view in the version used, although this is in agreement with most previous studies and is considered adequate under current recommendations [21].

We did not compare with nuclear magnetic resonance, given the limited availability of the technique in our centre and the large number of subjects.

We recognise that a comparative analysis between the three different methods, fully automatic, semi-automatic and manual, could be of interest. However, this has already been done previously [5]. In addition, the fully automatic method is considered less reliable. Since our aim was not to validate the software but to provide useful information on feasibility and reproducibility and to make available strain values in the paediatric population in our environment, we decided not to include such a comparation in our study.

Finally, the correlation R-values are very low for most comparisons. In addition, much of the data, although they show few statistical differences, are not clinically important; given the weak relationship we found between the correlation coefficient strain values and age and BSA, we decided not to perform Z-score analysis for nomogram construction as other studies have done.

## 5. Conclusions

We report that the analysis of the left ventricle, the right ventricle and the left atrium is highly accurate and feasible using this new software in semi-automatic mode. In a discrete manner, the strain values for all cardiac chambers present relationships with age, BSA, and sex, especially for the right ventricle. Left atrial strain values are less replicable than the ventricular strain values and are related to atrial volumes and parameters affecting left ventricular diastolic function.

## Figures and Tables

**Table 1:** The demographic, anthropometric and conventional echocardiographic parameters categorized by sex.

Variables	Total (N:156)	Females (N:78)	Males (N:78)	*p* Value
**BASELINE CHARACTERISTICS N ± SD**				
**Age (years)**	11 ± 3	11 ± 3	12 ± 3	0.23
**Weight (Kg)**	38 ± 13	37 ± 13	39 ± 13	0.2
**Height (cm)**	142 ± 16	139 ± 17	144 ± 17	0.059
**BSA (m^2^)**	1.2 ± 0.27	1.19 ± 0.27	1.25 ± 0.28	0.17
**BMI (Kg/m^2^)**	18 ± 2.7	19 ± 2.9	18 ± 2.5	0.4
**SBP (mmHg)**	108 ± 10	106 ± 10	110 ± 10	**0.028**
**CONVENTIONAL ECHOCARDIOGRAPHIC FINDINGS**				
**LVEF (%)**	65 ± 4	65 ± 5	66 ± 3	0.25
**EDV (mL)**	60 ± 18	54 ± 16	66 ± 19	**<0.0001**
**EDV Index (mL/m²)**	49.5 ± 8.4	46.4 ± 7.6	52.5 ± 8	**<0.0001**
**LV Mass (gr)**	72 ± 29	64 ± 21	80 ± 34	**0.001**
**LV Mass index (gr/m²)**	58.6 ± 15.2	54.2 ± 11.6	63 ± 17.1	**0.001**
**A Velocity (cm/s)**	58 ± 13	61 ± 13	55 ± 12	**0.002**
**E/A Ratio**	1.9 (1.6–2.1)	1.8 (1.6–2)	1.9 (1.6–2.1)	**0.005**
**E/e’ Ratio**	6.4 ± 1.1	6.4 ± 1.1	6.3 ± 1.1	0.48
**LA Vol (mL)**	21 ± 7	19 ± 5	23 ± 7	**0.001**
**LA Vol Index (mL/m²)**	17.3 ± 4.6	16.4 ± 4.5	18.2 ± 4.5	**0.019**
**TAPSE (mm)**	21 ± 3.8	21 ± 4	21 ± 3	0.56
**FAC (%)**	50 ± 8	50 ± 8	49 ± 8	0.76
**TEI index**	0.75 ± 0.06	0.76 ± 0.07	0.74 ± 0.06	0.24
**S´ RV (cm/s)**	13.7 ± 2.2	14 ± 2.3	14 ± 2.3	0.54
**RVBD (mm)**	28 ± 4	26 ± 4	29 ± 4	**<0.0001**

BSA: body surface area, BMI: body mass index, SBP: systolic blood pressure, LVEF: left ventricular ejection fraction biplane by Simpson, EDV: end diastolic volume, LV: left ventricle, LA: left atrium, TAPSE: tricuspid annular plane systolic excursion, SD: standard deviation, FAC: right ventricular fractional area change, RVBD: right ventricle basal diameter. Values are expressed as no. (%), or median ± standard deviation. Bold values indicate *p*-values < 0.05.

**Table 2:** The demographic, anthropometric and conventional echocardiographic parameters categorized by age.

Variables	6–9 Years (N:62)	10–12 Years (N:48)	13–17 Years (N:46)	*p* Value
**BASELINE CHARACTERISTICS**				
**Male (%)**	19 (44)	24 (54)	37 (54)	0.86
**Weight (Kg)**	27 ± 5	40 ± 9	51 ± 10	**<0.0001**
**Height (cm)**	126 ± 7	145 ± 9	159 ± 8	**<0.0001**
**BSA (m^2^)**	0.97 ± 0.12	1.27 ± 0.19	1.50 ± 0.19	**<0.0001**
**BMI (Kg/m^2^)**	16.8 ± 1.8	18.9 ± 2.6	20.1 ± 2.6	**<0.0001**
**SBP (mmHg)**	103 ± 8	108 ± 10	115 ± 8	**<0.0001**
**CONVENTIONAL ECHOCARDIOGRAPHIC FINDINGS**				
**LVEF (%)**	65 ± 4	65 ± 5	66 ± 4	0.45
**EDV (mL)**	47 ± 11	62 ± 12	77 ± 17	**<0.0001**
**EDV Index (mL/m²)**	48 ± 8	49 ± 7	51 ± 9	0.227
**Mass LV (gr)**	55 ± 18	72 ± 20	95 ± 34	**<0.0001**
**LV Mass index (gr/m²)**	56 ± 15	56 ± 11	63 ± 19	**0.047**
**A Vel (cm/s)**	61 ± 12	58 ± 14	56 ± 12	0.142
**E/e´ Ratio**	6.7 ± 1.1	6.3 ± 1.1	6.4 ± 1.1	**0.003**
**LA Volume (mL)**	18 ± 5	20 ± 6	25 ± 8	**<0.0001**
**LA Vol Index (mL/m²)**	19 ± 5	16 ± 4	17 ± 4	**0.016**
**TAPSE (mm)**	20 ± 4	22 ± 3	23 ± 3	**<0.0001**
**FAC (%)**	52 ± 7	48 ± 9	50 ± 8	**0.044**
**TEI index**	0.75 ± 0.06	0.74 ± 0.08	0.76 ± 0.05	0.68
**S´ RV (cm/s)**	13 ± 2.2	14 ± 2.3	14 ± 2.1	**0.022**
**RV basal (mm)**	26 ± 4	28 ± 4	30 ± 4	**<0.0001**

BSA: body surface area, BMI: body mass index, SBP: systolic blood pressure, LVEF: left ventricular ejection fraction biplane by Simpson, EDV: end diastolic volume, LV: left ventricle, LA: left atrium, TAPSE: tricuspid annular plane systolic excursion, SD: standard deviation, FAC: right ventricular fractional area change, RVBD: right ventricle basal diameter. Values are expressed as no. (%), or median ± standard deviation. Bold values indicate *p*-values < 0.05.

**Table 3:** The feasibility of the strain analysis categorized by age and sex.

Variables	Total (N:156)	6–9 Years (N:62)	10–12 Years (N:48)	13–17 Years (N:46)	*p* Value	Females (N:78)	Males (N:78)	*p* Value
**LVGLS (%)**	147 (94.2)	61 (98.4)	42 (87.5)	44 (95.7)	**0.046**	72 (92.3)	75 (96.2)	0.45
**RVFWLS (%)**	152 (97.4)	62 (100)	45 (93.8)	45 (97.8)	0.120	75 (96.2)	77 (98.7)	0.62
**RV4CLS (%)**	153 (98.1)	62 (100)	45 (93.8)	46 (100)	**0.032**	76 (97.4)	77 (98.7)	0.56
**LALSr (%)**	153 (98.1)	62 (100)	46 (95.8)	45 (97.8)	0.289	76 (97.4)	77 (98.7)	0.56
**LALScd (%)**	153 (98.1)	62 (100)	46 (95.8)	45 (97.8)	0.289	76 (97.4)	77 (98.7)	0.56
**LALSct (%)**	152 (97.4)	62 (100)	45 (93.8)	45 (97.8)	0.120	75 (96.2)	77 (98.7)	0.62

LVGLS: Left ventricle global longitudinal strain, RVFWLS: Right ventricle free wall longitudinal strain RV4CLS: Right ventricle four chamber longitudinal strain, LALSr: Left atrial longitudinal strain reservoir phase, LALScd: Left atrial longitudinal strain conduct phase, LALSct: Left atrial longitudinal strain contraction phase. Values are expressed as no. (%). Bold values indicate *p*-values < 0.05.

**Table 4:** Strain values categorized by sex.

Variables	Total (N:153)	Females (N:76)	Males (N:77)	*p* Value
**LVGLS**	26.7 ± 2.3	27 ± 1.9	26.3 ± 2.5	**0.041**
**RVFWLS**	30.5 ± 4.4	32 ± 4.4	29.1 ± 3.9	**<0.0001**
**RV4CLS**	26.9 ± 4	28.1 ± 4.1	25.7 ± 3.5	**<0.0001**
**LALSr**	57.8 ± 10.5	59.7 ± 9.7	56 ± 11.1	**0.032**
**LALScd**	44.9 ± 9.5	45.8 ± 8.3	44 ± 10.7	0.26
**LALSct**	12.9 ± 5.5	13.9 ± 6	12 ± 4.7	**0.03**

LVGLS: Left ventricle global longitudinal strain, RVFWLS: Right ventricle free wall longitudinal strain RV4CLS: Right ventricle four chamber longitudinal strain, LALSr: Left atrial longitudinal strain reservoir phase, LALScd: Left atrial longitudinal strain conduct phase, LALSct: Left atrial longitudinal strain contraction phase. Values are expressed as no. (%). Bold values indicate *p*-values < 0.05.

**Table 5:** Strain values categorized by age groups.

Variables	Group 1 (N:62) 6–9 Years	Group 2 (N:48) 10–12 Years	Group 3 (N:46) 13–17 Years	*p* Value	P1 *	P2 *	P3 *
**LVGLS**	27.3 ± 2.1	26.2 ± 2.7	23.3 ± 2.25	**0.016**	0.07	**0.021**	0.99
**RVFWLS**	32.6 ± 3.7	29.8 ± 4.3	28.3 ± 4.1	**<0.0001**	**0.002**	**<0.0001**	0.22
**RV4CLS**	28.7 ± 3.2	26.4 ± 4.2	24.9 ± 3.7	**<0.0001**	**0.005**	**<0.0001**	0.18
**LALSr**	60.2 ± 9.4	57.6 ± 12.3	54.8 ± 9.5	**0.031**	0.45	**0.012**	0.45
**LALScd**	47.3 ± 8.9	43.9 ± 10.4	42.7 ± 8.9	**0.038**	0.21	**0.049**	1
**LALSct**	13 ± 5.4	13.7 ± 5.6	12 ± 5.3	0.35	1	1	0.44

LVGLS: Left ventricle global longitudinal strain, RVFWLS: Right ventricle free wall longitudinal strain RV4CLS: Right ventricle four chamber longitudinal strain, LALSr: Left atrial longitudinal strain reservoir phase, LALScd: Left atrial longitudinal strain conduct phase, LALSct: Left atrial longitudinal strain contraction phase. Values are expressed as median ± standard deviation. Bold values indicate *p*-values < 0.05. * Post hoc Bonferroni or Games-Howell correction. P1: group 1 vs. 2, P2: group 1 vs. 3 and P3: group 2 vs. 3.

**Table 6:** Strain values categorized by body surface area groups.

Variables	Group 1 (N:62) 0.75–1.02 m^2^	Group 2 (N:48) 1.03–1.35 m^2^	Group 3 (N:46) 1.36–1.87 m^2^	*p* Value	P1 *	P2 *	P3 *
**LVGLS**	27.1 ± 2.3	26.8 ± 2.5	26.16 ± 1.99	0.112	1	0.117	0.56
**RVFWLS**	32.4 ± 4.2	31.2 ± 3.9	28 ± 3.9	**<0.0001**	0.37	**<0.0001**	**<0.0001**
**RV4CLS**	28.5 ± 3.6	27.5 ± 4	24.7 ± 3.3	**<0.0001**	0.52	**<0.0001**	**<0.0001**
**LALSr**	60.4 ± 10.5	58 ± 10.3	55.2 ± 10.4	**0.043**	0.74	**0.038**	0.5
**LALScd**	47 ± 0.5	44.7 ± 9.4	43.1 ± 9.5	0.112	0.64	0.11	1
**LALSct**	13.4 ± 5.4	13.3 ± 5.3	12.1 ± 5.6	0.45	1	0.79	0.87

LVGLS: Left ventricle global longitudinal strain, RVFWLS: Right ventricle free wall longitudinal strain RV4CLS: Right ventricle four chamber longitudinal strain, LALSr: Left atrial longitudinal strain reservoir phase, LALScd: Left atrial longitudinal strain conduct phase, LALSct: Left atrial longitudinal strain contraction phase. Values are expressed as median ± standard deviation. Bold values indicate *p*-values < 0.05. * Post hoc Bonferroni or Games-Howell correction. P1: group 1 vs. 2, P2: group 1 vs. 3 and P3: Group 2 vs. 3.

**Table 7:** The univariate and multivariate regression model results for left ventricle strain.

Variables	R^2^	*p* Value	B Unstandardised	*p* Value
**Age**	−0.2	0.01		
**BSA**	−0.24	**0.002**		
**SBP**	−0.22	**0.005**		
**LV Mass**	−0.22	**0.005**		
**DDLV**	−0.2	0.01		
**EDV**	−0.25	**0.001**	−0.032	**0.001**
**LVEF**	0.2	**0.009**	0.117	**0.001**

R^2^: Determination coefficient, BSA body surface area, SBP systolic blood pressure, LV left ventricle, DDLV end diastolic diameter of left ventricle, EDV end diastolic volume, LVEF left ventricular ejection fraction biplane by Simpson. Variables that reached *p*-values < 0.01 in the univariate model and did not show collinearity with other variables (VIF < 5) were included in the multivariate model. In cases of collinearity, the variable with the highest correlation coefficient was included in the multivariate model. If a variable included in the multivariate model did not reach statistical significance, it was excluded by backward steps. r = 0.322, R^2^ = 0.104 and corrected 0.091 for the multivariate regression model, *p*: 0.001. Bold value indicates *p*-values < 0.01.

**Table 8:** The univariate and multivariate regression model results for right ventricle strain.

Variables	RVFWLS				RV4CLS			
	R^2^	*p* Value	B Unstandardised	*p* Value	R^2^	*p* Value	B Unstandardised	*p* Value
**Age**	−0.42	**<0.0001**			−0.4	**<0.0001**	−0.45	**<0.0001**
**BSA**	−0.42	**<0.0001**	−9.655	**<0.0001**	−0.38	**<0.0001**		
**Sex**	−0.35	**<0.0001**	−2.794	**<0.0001**	−0.32	**<0.0001**	−1.98	**<0.0001**
**LV Mass**	−0.24	**0.002**	0.048	**<0.0001**	−0.27	**0.001**		
**DDLV**	−0.38	**<0.0001**			−0.34	**<0.0001**		
**EDV**	−0.37	**<0.0001**			−0.34	**<0.0001**		
**SBP**	−0.15	0.039						
**RV basal**	−0.28	**<0.0001**			−0.28	**<0.0001**		
**RVDA**	−0.32	**<0.0001**			−0.28	**<0.0001**		
**A Vel**	0.22	**0.004**			−0.1	0.049		
**TAPSE**	−0.2	0.01						
**LA Vol**	−0.15				−0.21	**0.008**		

RVFWLS: Right ventricle free wall longitudinal strain, RV4CLS: Right ventricle four-chamber longitudinal strain, R^2^: Determination coefficient, BSA: body surface area, LV: left ventricle, DDLV: end diastolic diameter of left ventricle, EDV: end diastolic volume, SBP: systolic blood pressure, RV: right ventricle, RVDA: right ventricle diastolic area, TAPSE: tricuspid annular plane systolic excursion, LA: left atrial. Variables that reached *p*-values < 0.01 in the univariate model and did not show collinearity with other variables (VIF < 5) were included in the multivariate model. In case of collinearity, the variable with the highest correlation coefficient was included in the multivariate model. If a variable included in the multivariate model did not reach statistical significance, it was excluded by backward steps. In the RVFWLS multivariate regression model, r = 0.540, R^2^ = 0.291 and corrected R^2^ = 0.276, *p* < 0.0001. In the RV4CLS multivariate regression model, r = 0.477, R^2^ = 0.227 and corrected R^2^ = 0.215, *p* < 0.0001. Bold values indicate *p*-values < 0.01.

**Table 9:** The univariate and multivariate regression model results for left atrial strain.

Variables	LALSr				LALScd				LALSct			
	R^2^	*p* Value	Beta	*p* Value	R^2^	*p* Value	Beta	*p* Value	R^2^	*p* Value	Beta	*p* Value
**Age**	−0.21	**0.008**			−0.21	**0.009**						
**BSA**	−0.21	**0.008**			−0.19	0.018						
**Sex**	−0.2	0.011							−0.18	0.019		
**LV mass**	−0.27	**0.001**			−0.27	**0.001**	−0.86	**0.002**				
**DDLV**	−0.24	**0.003**			−0.2	**0.009**						
**EDV**	−0.24	**0.002**			−0.25	**0.002**						
**RV basal**	−0.25	**0.002**			−0.2	0.01						
**RVDA**	−0.19	0.016							0.31	0.031		
**A Vel**	0.17	0.026			0.05				0.27	**0.001**	0.113	**0.002**
**LA Vol**	−0.3	**<0.0001**	−0.465	**<0.0005**	−0.25	**0.002**			−0.12			
**TDV A septal**									0.24	**0.003**		
**E/A Ratio**									−0.18	0.018		

LALSr: Left atrial longitudinal strain reservoir phase, LALScd: Left atrial longitudinal strain conduct phase, LALSct: Left atrial longitudinal strain contraction phase, R^2^: Determination coefficient, BSA: body surface area, LV: left ventricle, DDLV: end diastolic diameter of left ventricle, EDV: end diastolic volume, RV: right ventricle, RVDA: right ventricle diastolic area, LA: left atrial, TDV: tissue doppler velocity. Variables that reached *p*-values < 0.01 in the univariate model and did not show collinearity with other variables (VIF < 5) were included in the multivariate model. In cases of collinearity, the variable with the highest correlation coefficient was included in the multivariate model. If a variable included in the multivariate model did not reach statistical significance, it was excluded by backward steps. In the LALSr multivariable regression model, r = 0.299, R^2^ = 0.089 and corrected R^2^ = 0.083 *p* < 0.0005. In the LALScd multivariable regression model, r = 0.270, R^2^ = 0.073 and corrected R^2^ = 0.066 *p* < 0.002. In the LALScp multivariable regression model, r = 0.272, R^2^ = 0.074 and corrected R^2^ = 0.067, *p* < 0.002. Bold values indicate *p*-values < 0.01.

**Table 10:** The inter- and Intra-observer reproducibility analysis results.

Intra-Observer	ICC (95% CI)	*p* Value	Bland-Altman Bias (LOA)	*p* Value
**LVGLS**	0.977 (0.942–0.991)	<0.0001	−0.1 (−1.45 to 1.35)	0.2
**RVFWLS**	0.953 (0.882–0.982)	<0.0001	0.055 (−3.34 to 3.45)	0.433
**RV4CLS**	0.927 (0.815–0.971)	<0.0001	0.01 (−3.80 to 3.82)	0.827
**LALSr**	0.859 (0.533–0.950)	<0.0001	3.025 (−5.76 to 11.81)	0.511
**LALScd**	0.756 (0.315–0.907)	<0.0001	3.305 (−6.86 to 13.47)	0.774
**LALScp**	0.792 (0.471–0.918)	0.001	−0.46 (−8.54 to 7.62)	0.824
**Inter-observer**				
**LVGLS**	0.924 (0.809–0.970)	<0.0001	−0.17 (−2.55 to 2.39)	0.497
**RVFWLS**	0.920 (0.795–0.968)	<0.0001	0.050 (−4.14 to 4.24)	0.995
**RV4CLS**	0.882 (0.707–0.953)	<0.0001	−0.75 (−5.41 to 3.91)	0.918
**LALSr**	0.734 (0.114–0.907)	<0.0001	4.639 (−6.07 to 15.35)	0.302
**LALScd**	0.778 (0.367–0.917)	<0.0001	3.3 (−6.94 to 13.54)	0.775
**LALSct**	0.719 (0.311–0.887)	0.004	−1.21 (−9.27 to 6.85)	0.152

ICC: intraclass correlation coefficient, LOA: 95% limit of agreement, LVGLS: Left ventricle global longitudinal strain, RVFWLS: Right ventricle free wall longitudinal strain RV4CLS: Right ventricle four-chamber longitudinal strain, LALSr: Left atrial longitudinal strain reservoir phase, LALScd: Left atrial longitudinal strain conduct phase, LALSct: Left atrial longitudinal strain contraction phase.

## Abbreviations

LVGLS: Left ventricle global longitudinal strain, RVFWLS: Right ventricle free wall longitudinal strain, RV4CLS: Right ventricle four-chamber longitudinal strain, LALSr: Left atrial longitudinal strain reservoir phase, LALScd: Left atrial longitudinal strain conduct phase, LALSct: Left atrial longitudinal strain contraction phase.
